# Author Correction: Airway response to respiratory syncytial virus has incidental antibacterial effects

**DOI:** 10.1038/s41467-019-11222-9

**Published:** 2019-07-18

**Authors:** Charles J. Sande, James M. Njunge, Joyce Mwongeli Ngoi, Martin N. Mutunga, Timothy Chege, Elijah T. Gicheru, Elizabeth M. Gardiner, Agnes Gwela, Christopher A. Green, Simon B. Drysdale, James A. Berkley, D. James Nokes, Andrew J. Pollard

**Affiliations:** 1 KEMRI-Wellcome Trust Research Programme, Bofa Rd, Kilifi, - P.O. Box 230 - 80108 Kenya; 2 Oxford Vaccine Group, University of Oxford, and the NIHR Oxford Biomedical Research Centre, Oxford, Oxford, OX3 7LE UK; 3 Centre for Tropical Medicine and Global Health, Nuffield Department of Medicine, University of Oxford, OX3 7FZ Oxford, UK; 4 The Childhood Acute Illness & Nutrition (CHAIN) Network, Nairobi, - P.O. Box 43640-00100 Kenya; 5 School of Life Sciences and Zeeman Institute (SBIDER), University of Warwick, CV4 7AL Coventry, UK

**Keywords:** Proteomics, Infectious diseases, Mucosal immunology, Infection

Correction to: *Nature Communications* 10.1038/s41467-019-10222-z, published online 17 May 2019.

The original version of this Article contained an error in the spelling of the author D. James Nokes, which was incorrectly given as James Nokes. This has now been corrected in both the PDF and HTML versions of the Article.

