# Supplementary material for: Airway response to respiratory syncytial virus has incidental antibacterial effects
Source: Nat Commun. 2019 May 17;10:2218. doi: 10.1038/s41467-019-10222-z (PMC6525170; doi:10.1038/s41467-019-10222-z)
Supplement: Supplementary file 1 — Supplementary Information [file 41467_2019_10222_MOESM1_ESM.pdf]

Supplementary information

Airway response to respiratory syncytial virus has incidental antibacterial effects

Sande et al.

Supplementary figures

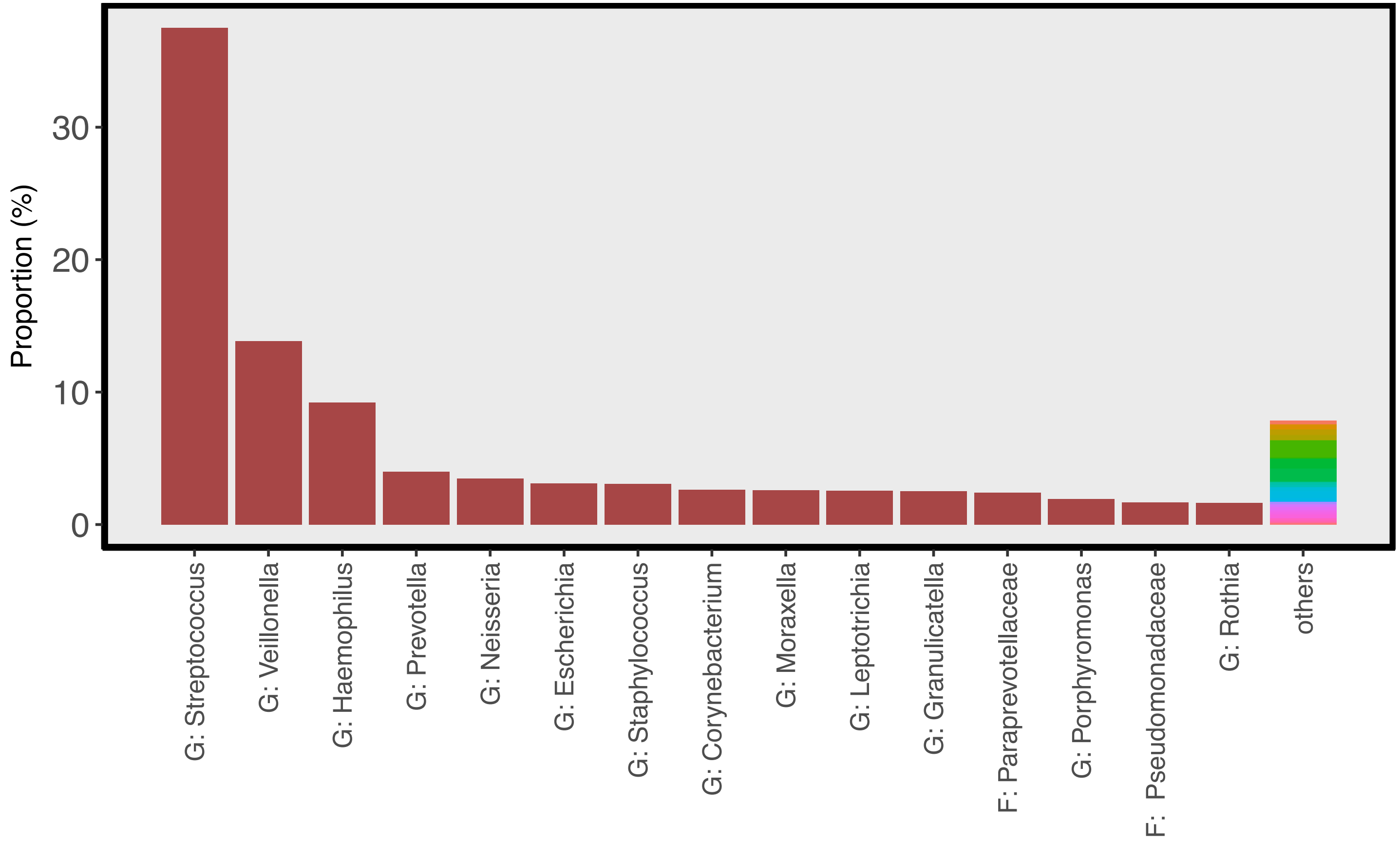

Supplementary figure 1**. Diversity of airway microbiota**. The relative proportional abundances of the top 15 bacterial OTUs in the study population of 84 children are shown. The most abundant bacterial taxa was Streptococcus, with a median proportional abundance of ~40%. Source data are provided as a Source Data file.

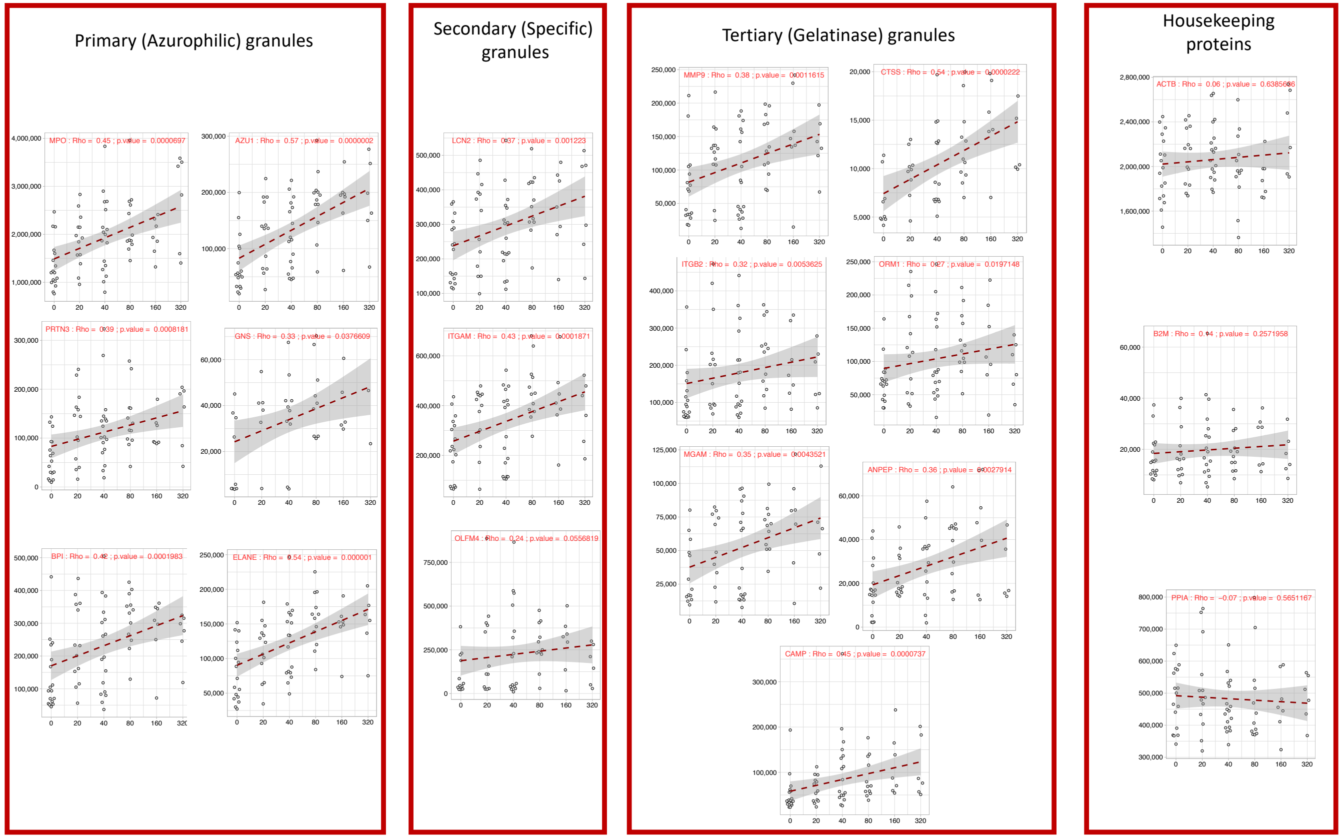

Supplementary figure 2: **Correlation between airway neutrophil granule protein expression and bacterial inhibition activity**. The expression levels of 16 neutrophil granule – stratified into their respective functional subsets i.e. primary (azurophilic), secondary (specific) and tertiary (gelatinase) – were correlated with bacterial inhibition activity in nasal secretions measured using an *in vitro* bacterial inhibition assay. A strong, statistically significant positive correlation was observed between granule expression and inhibition activity. However no correlation was observed when the expression levels of housekeeping proteins (ACTB, B2M and PPIA) was compared to inhibition activity. Correlation analysis was done using Spearman's rank order correlation. Source data are provided as a Source Data file.

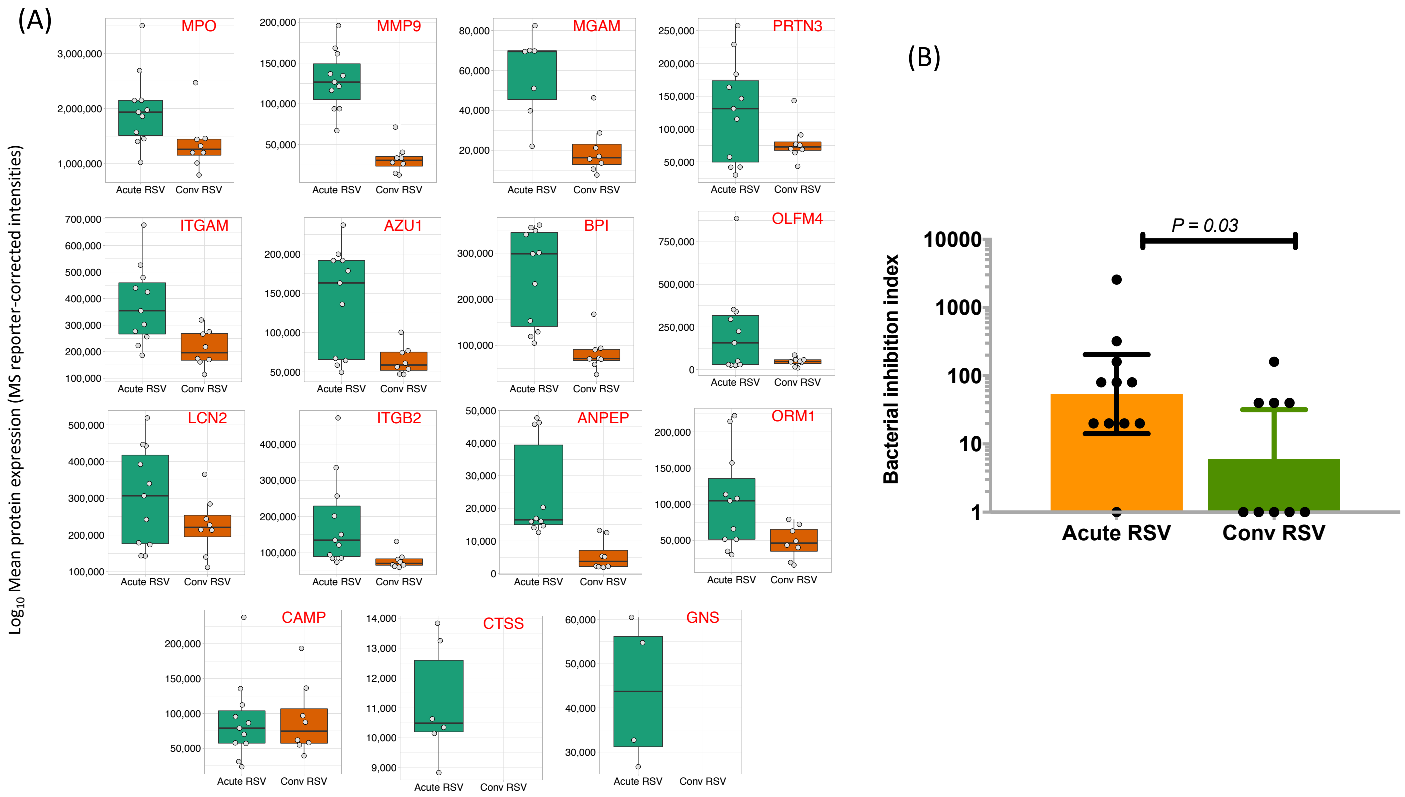

Supplementary figure 3: **Analysis of bacterial inhibition during acute disease and at convalescence**. For most neutrophil granule proteins, relative expression levels at the acute time point were greater relative to the convalescent time point (one month later). Correspondingly, airway secretions collected at the acute time point, had significantly greater bacterial inhibition activity compared to those collected at convalescence. Statistical differences between bacterial inhibition at the acute and convalescent time points were measured using the Mann-Whitney U test. The elements of the box and whisker plots are defined in figure 1. Source data are provided as a Source Data file.

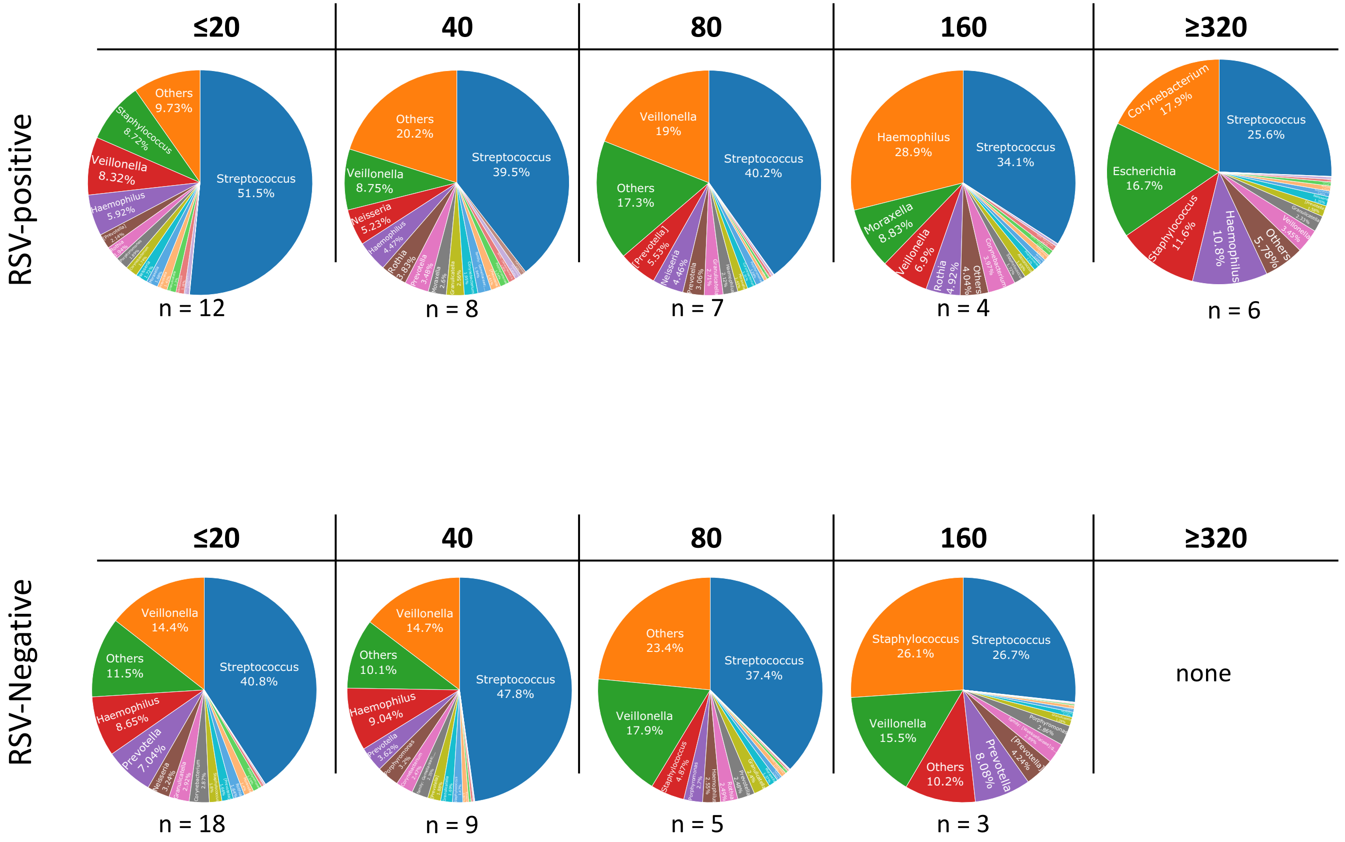

Supplementary figure 4. **Comparative analysis of bacterial inhibition activity in RSV-positive and RSV-negative children**. Bacterial inhibition indices in RSV-positive and RSV negative children were compared with 6 strata shown. In general, the airway abundance of Streptococcus declined with an increase in the inhibition index. Source data are provided as a Source Data file.

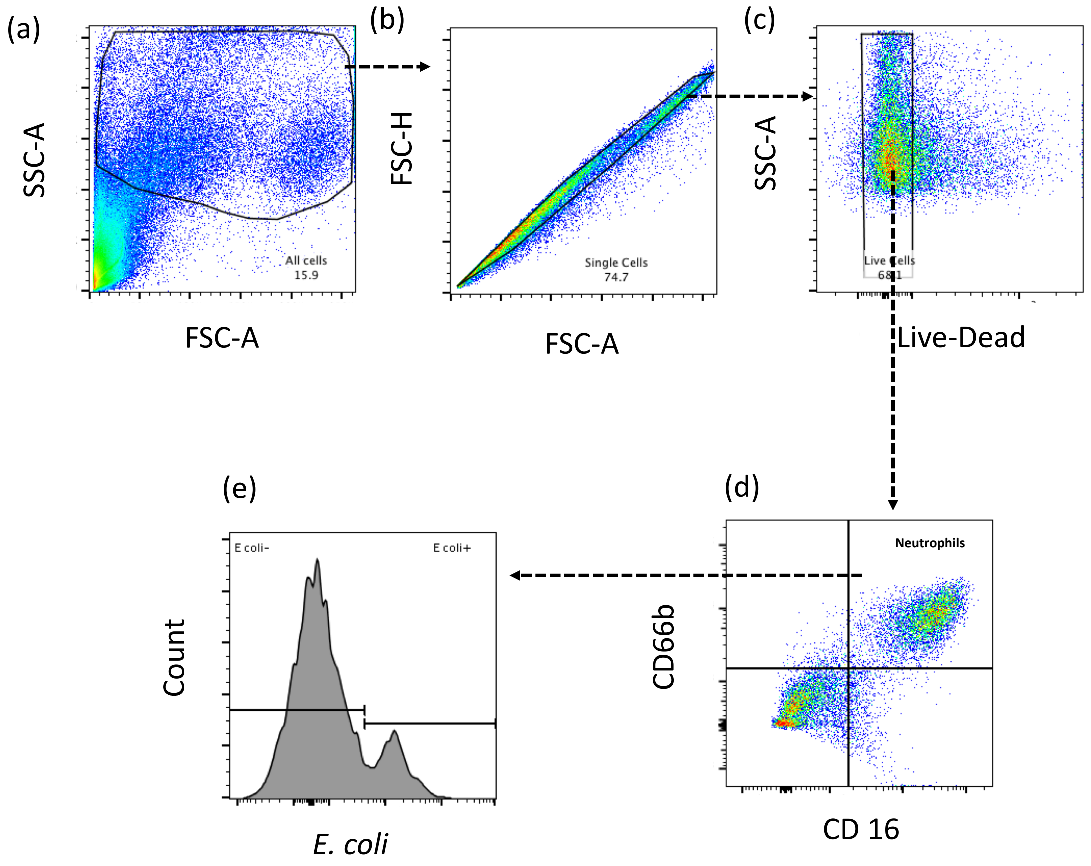

Supplementary figure 5. **Gating strategy used to identify airway neutrophils**. Neutrophils were defined as live, singlet cells that were double positive for CD66b+, CD16+ (A-D). Neutrophil phagocytosis was measured by expression of the pHrodo dye, whose florescence was only activated upon ingestion of labelled E. coli into the neutrophil phagosome(E).

Supplementary methods

Analysis of the airway proteome by high performance liquid chromatography-tandem mass spectrometry (HPLC-MS/MS)

Airway samples were centrifuged at 17,000xg for 10 mins at 4°C to obtain cell pellets which were washed once using PBS and lysed by bead-vortexing for 10 minutes in cell lysis buffer (RLT, Qiagen, Germany). Proteins (as well as DNA and RNA) were then extracted from the lysate using the AllPrep DNA/RNA/Protein Mini Kit (Qiagen, Germany) following manufacturers instructions. The concentration of total protein obtained was determined using the Bradford assay (Bio-Rad, USA). Thirty micrograms (30µg) of total protein from each sample was then reduced with 10mM tris(2-carboxyethyl)phosphine (TCEP, Sigma-Aldrich, USA) at 55°C for 1h and subsequently alkylated with 18mM IAA (Sigma-Aldrich, USA) for 30 minutes at room temperature, while keeping the reaction protected from light. Proteins were precipitated overnight at -20°C with six volumes of pre-chilled (-20°C) acetone (Sigma-Aldrich, USA). The samples were centrifuged at 8,000xg for 10 minutes at 4°C to obtain the protein pellets and supernatants were discarded. The protein pellet was resuspended in 100µl of 50mM Triethylammonium bicarbonate (TEAB, Sigma-Aldrich, USA). Trypsin (Sigma-Aldrich, USA) was added to the protein samples at a trypsin-protein sample ratio of 1:10 and protein digestion was allowed to proceed overnight at 37°C with shaking. The peptide samples were randomly assigned to 10 individual batches: each containing nine patient samples and one pooled control sample. The pooled control sample consisted of a pool of peptides from all patient samples. The peptide samples derived from individual patients were then individually labelled using the TMT10plex mass tag kit (Thermo scientific, USA) according to manufacturer's instructions, with one isobaric tag being exclusively used to label the pooled

control sample. The labelled peptides for each 10plex were subsequently combined to generate 10 individual pools. The labelled peptide pools were desalted using P10 C18 pipette ZipTips (Millipore, USA) according to the manufacturer's instructions. Eluted peptides were dried in a Speedvac concentrator (Thermo Scientific, USA). Peptides (8 µl) were loaded using a Dionex Ultimate 3000 nano-flow ultra-high-pressure liquid chromatography system (Thermo Scientific, USA) on to a 75µm x 2 cm C18 trap column (Thermo Scientific, USA) and separated on a 75µm x 50 cm C18 reverse-phase analytical column (Thermo Scientific) at heated at 40°C. For LFQ protein quantification; elution was carried out with mobile phase B (80% acetonitrile with 0.1% formic acid) gradient (4 to 30%) over 310 min at a flow rate of 0.25 µl/min. Each LC run was finished by washout with 98% B for 10 min and re-equilibration in 2% B for 30 min. Five blanks of 40 min each were run on the column between each injection comprising of two wash cycles with 90% B and an equilibration phase of 15 min to avoid sample carryover. Peptides were measured using a Q Exactive Orbitrap mass spectrometer (Thermo Scientific, USA) coupled to the chromatography system via a nano-electrospray ion source (Thermo Scientific). On the Q Exactive , the ms^1 settings for peptides were: Resolution, 70000; AGC target, 3e6; maximum IT, 120 ms; scan range, 400-1800 m/z; while the ms^2 settings for fragmentation spectra of peptides were: Resolution, 17000 (35000 for labelled peptides); AGC target, 5e4; maximum IT, 120 ms ; isolation window, 1.6 m/z. MS data were acquired by data dependent acquisition where the top 12 (15 for labelled peptides) most intense precursor ions in positive mode were selected for ms^2 Higher-energy C-trap dissociation fragmentation which were subsequently excluded for the next 45 s following fragmentation event. Charge exclusion was set to ignore peptide spectrum matches that were unassigned, singly charged, and those with ≥+8 charges. Raw mass spectrometer files were

analysed by MaxQuant software version 1.6.0.1. by searching against the human Uniprot FASTA database (downloaded February 2014) using the Andromeda search engine.
