# Supplementary material for: Airway response to respiratory syncytial virus has incidental antibacterial effects
Source: Nat Commun. 2019 May 17;10:2218. doi: 10.1038/s41467-019-10222-z (PMC6525170; doi:10.1038/s41467-019-10222-z)
Supplement: Supplementary file 3 — Description of Additional Supplementary Files [file 41467_2019_10222_MOESM3_ESM.docx]

**Description of Supplementary Files**

**File Name:** Supplementary Data 1

**Description:** The taxonomic identities of low-abundance (<1%) operational taxonomic units.

**File Name:** Supplementary Data 2

**Description:** List of 123 airway proteins that were differentially expressed between RSV-positive and RSVnegative children.
