# Supplementary material for: Airway response to respiratory syncytial virus has incidental antibacterial effects
Source: Nat Commun. 2019 May 17;10:2218. doi: 10.1038/s41467-019-10222-z (PMC6525170; doi:10.1038/s41467-019-10222-z)
Supplement: Supplementary file 6 — Reporting Summary [file 41467_2019_10222_MOESM6_ESM.pdf]

nature research

Corresponding author(s): *Double-blind peer review submissions: write DBPR and your manuscript number here instead of author names.*

Last updated by author(s): *YYYY-MM-DD*

Reporting Summary

Nature Research wishes to improve the reproducibility of the work that we publish. This form provides structure for consistency and transparency in reporting. For further information on Nature Research policies, see Authors & Referees and the Editorial Policy Checklist.

Statistics

For all statistical analyses, confirm that the following items are present in the figure legend, table legend, main text, or Methods section.

n/a	Confirmed	
☐	☒	The exact sample size (*n*) for each experimental group/condition, given as a discrete number and unit of measurement
☐	☒	A statement on whether measurements were taken from distinct samples or whether the same sample was measured repeatedly
☐	☒	The statistical test(s) used AND whether they are one- or two-sided<br>*Only common tests should be described solely by name; describe more complex techniques in the Methods section.*
☐	☒	A description of all covariates tested
☐	☒	A description of any assumptions or corrections, such as tests of normality and adjustment for multiple comparisons
☐	☒	A full description of the statistical parameters including central tendency (e.g. means) or other basic estimates (e.g. regression coefficient) AND variation (e.g. standard deviation) or associated estimates of uncertainty (e.g. confidence intervals)
☐	☒	For null hypothesis testing, the test statistic (e.g. *F*, *t*, *r*) with confidence intervals, effect sizes, degrees of freedom and *P* value noted<br>*Give P values as exact values whenever suitable.*
☒	☐	For Bayesian analysis, information on the choice of priors and Markov chain Monte Carlo settings
☒	☐	For hierarchical and complex designs, identification of the appropriate level for tests and full reporting of outcomes
☒	☐	Estimates of effect sizes (e.g. Cohen's *d*, Pearson's *r*), indicating how they were calculated

Our web collection on statistics for biologists contains articles on many of the points above.

Software and code

Policy information about availability of computer code

Data collection	Flow cytometry data on the LSR Fortessa were collected using FACSDiva software, while proteomics data on the Q-exactive mass spectrometer was XCalibur software
Data analysis	Data were analyzed using R software for statistical computing - version 3.4.0 (2017-04-21) , FlowJo and MaxQuant software

For manuscripts utilizing custom algorithms or software that are central to the research but not yet described in published literature, software must be made available to editors/reviewers. We strongly encourage code deposition in a community repository (e.g. GitHub). See the Nature Research guidelines for submitting code & software for further information.

Data

Policy information about availability of data

All manuscripts must include a data availability statement. This statement should provide the following information, where applicable:

- Accession codes, unique identifiers, or web links for publicly available datasets
- A list of figures that have associated raw data
- A description of any restrictions on data availability

The proteomics data reported in this paper are available at the ProteomeXchange Consortium database (Accession number: PXD009403) while the microbiome data are available at the European Nucleotide Archive database (Accession number PRJEB28053). Figures were generated from these data sets. No restrictions apply in the use of these data.

Field-specific reporting

Please select the one below that is the best fit for your research. If you are not sure, read the appropriate sections before making your selection.

☒ Life sciences ☐ Behavioural & social sciences ☐ Ecological, evolutionary & environmental sciences

For a reference copy of the document with all sections, see nature.com/documents/nr-reporting-summary-flat.pdf

Life sciences study design

All studies must disclose on these points even when the disclosure is negative.

Sample size	In this study all children who fulfilled a predefined diagnostic criteria for severe respiratory illness were included (i.e. children admitted to hospital with pneumonia and who did not have bacteraemia but were either positive for RSV and negative for other respiratory viruses (cases) or negative for all viruses(controls). All children who fulfilled this criteria (N=84) during the sampling period were included in the analysis.
Data exclusions	All children who fulfilled the criteria above were included and none were excluded
Replication	We used a large number of biological replicates in each group (RSV-positive and RSV-negative) in order to confirm that findings were reproducible with each phenotype. For the RSV-positive group, samples from 40 individual children were used while 44 individual children were sampled in the RSV-negative group
Randomization	Children were allocated to the RSV-positive group only if they had a laboratory confirmed diagnosis of RSV. Children were assigned to the RSV negative group if their laboratory tests for RSV were negative. Apart from the diagnostic criteria no further selection criteria were applied.
Blinding	The research team who undertook analysis of samples in the lab were blinded to whether children were in the RSV-positive or RSV-negative classification. However, the statistical analysis team had access to this information.

Reporting for specific materials, systems and methods

We require information from authors about some types of materials, experimental systems and methods used in many studies. Here, indicate whether each material, system or method listed is relevant to your study. If you are not sure if a list item applies to your research, read the appropriate section before selecting a response.

Materials & experimental systems

n/a	Involved in the study	
☒	☐	Antibodies
☒	☐	Eukaryotic cell lines
☒	☐	Palaeontology
☒	☐	Animals and other organisms
☐	☒	Human research participants
☒	☐	Clinical data

Methods

n/a	Involved in the study	
☒	☐	ChIP-seq
☐	☒	Flow cytometry
☒	☐	MRI-based neuroimaging

Human research participants

Policy information about studies involving human research participants

Population characteristics	The following covariates were available for the study participants (and are detailed in Table 1): Age, weight, length, oxygen saturation, respiratory rate and axillary temperature. Each of these variables was compared between RSV-positive and RSV-negative children and no statistically significant differences were found.
Recruitment	All children who were admitted to hospital with respiratory illness and fulfilled the diagnostic criteria for RSV set out above were included in the study. Since all eligible children were recruited and sampled and none were excluded the risk of selection bias is limited
Ethics oversight	Ethical approval for the conduct of this study was granted by the Kenya Medical Research Institute's Scientific and ethical research unit (SERU)

Note that full information on the approval of the study protocol must also be provided in the manuscript.

Flow Cytometry

Plots

Confirm that:

☒ The axis labels state the marker and fluorochrome used (e.g. CD4-FITC).

☒ The axis scales are clearly visible. Include numbers along axes only for bottom left plot of group (a 'group' is an analysis of identical markers).

☒ All plots are contour plots with outliers or pseudocolor plots.

☒ A numerical value for number of cells or percentage (with statistics) is provided.

Methodology

Sample preparation	1ml of nasopharyngeal and oropharyngeal swab samples obtained from children was centrifuged at 17,000xg for 7 minutes, after which 800⍰l of the supernatant was removed and discarded. The remaining 200⍰l were split into two aliquots of 100⍰l each. The first aliquot was used for neutrophil phenotyping assays and the other was used for neutrophil phagocytosis assays. 20 ⍰l of a pre-constituted cocktail of the following antibodies (from ThermoFisher) was used to label both aliquots – CD45, CD16, CD14, CD3, CD19, HLA-DR, CD66b, CD11b and a Live-dead marker.
Instrument	BD LSR Fortessa
Software	FlowJo
Cell population abundance	The abundance of airway neutrophils was determined on the basis of surface marker expression (CD16 and CD666b). The frequency of neutrophils that were taking up bacteria was characterised by the analysis of the expression level of E.coli labeled with a fluorescent tag that was only activated within a phagosome. The frequecies of these cells varied by group and individual - an example of the analysis of two individual samples is shown in figure 4.
Gating strategy	Granulocytes were selected and debris excluded on the basis of their forward (FSC-A) and side (SSC-A) scatter characteristics. Doublets were excluded using FSC-A versus FSC-H and dead cells were excluded using the live-dead marker. Cells that were double positive for CD66b and CD16 were gated as airway neutrophils

☒ Tick this box to confirm that a figure exemplifying the gating strategy is provided in the Supplementary Information.
